# Simple approach for the preparation of ^15−15^N_2_-enriched water for nitrogen fixation assessments: evaluation, application and recommendations

**DOI:** 10.3389/fmicb.2015.00769

**Published:** 2015-08-04

**Authors:** Isabell Klawonn, Gaute Lavik, Philipp Böning, Hannah K. Marchant, Julien Dekaezemacker, Wiebke Mohr, Helle Ploug

**Affiliations:** ^1^Department of Ecology, Environment and Plant Sciences, Stockholm UniversityStockholm, Sweden; ^2^Max Planck Institute for Marine MicrobiologyBremen, Germany; ^3^Institute of Chemistry and Biology of the Marine Environment, Carl von Ossietzky University of OldenburgOldenburg, Germany; ^4^Department of Marine Sciences, University of GothenburgGothenburg, Sweden

**Keywords:** N_2_ fixation, cyanobacteria, gas–liquid solution, ^15^N_2_ gas, gas solubility, iron, phosphorus, *Nodularia spumigena*

## Abstract

Recent findings revealed that the commonly used ^15^N_2_ tracer assay for the determination of dinitrogen (N_2_) fixation can underestimate the activity of aquatic N_2_-fixing organisms. Therefore, a modification to the method using pre-prepared ^15−15^N_2_-enriched water was proposed. Here, we present a rigorous assessment and outline a simple procedure for the preparation of ^15−15^N_2_-enriched water. We recommend to fill sterile-filtered water into serum bottles and to add ^15−15^N_2_ gas to the water in amounts exceeding the standard N_2_ solubility, followed by vigorous agitation (vortex mixing ≥ 5 min). Optionally, water can be degassed at low-pressure (≥950 mbar) for 10 min prior to the ^15−15^N_2_ gas addition to indirectly enhance the ^15−15^N_2_ concentration. This preparation of ^15−15^N_2_-enriched water can be done within 1 h using standard laboratory equipment. The final ^15^N-atom% excess was 5% after replacing 2–5% of the incubation volume with ^15−15^N_2_-enriched water. Notably, the addition of ^15−15^N_2_-enriched water can alter levels of trace elements in the incubation water due to the contact of ^15−15^N_2_-enriched water with glass, plastic and rubber ware. In our tests, levels of trace elements (Fe, P, Mn, Mo, Cu, Zn) increased by up to 0.1 nmol L^−1^ in the final incubation volume, which may bias rate measurements in regions where N_2_ fixation is limited by trace elements. For these regions, we tested an alternative way to enrich water with ^15−15^N_2_. The ^15−15^N_2_ was injected as a bubble directly to the incubation water, followed by gentle shaking. Immediately thereafter, the bubble was replaced with water to stop the ^15−15^N_2_ equilibration. This approach achieved a ^15^N-atom% excess of 6.6 ± 1.7% when adding 2 mL ^15−15^N_2_ per liter of incubation water. The herein presented methodological tests offer guidelines for the ^15^N_2_ tracer assay and thus, are crucial to circumvent methodological draw-backs for future N_2_ fixation assessments.

## Introduction

The availability of fixed nitrogen (N) in N-limiting habitats is a proximal driver of aquatic productivity and the subsequent sequestration of carbon dioxide (CO_2_) from the atmosphere to the sediment (Capone, 2001; Gruber and Galloway, 2008). Biological N_2_ fixation is the largest source of fixed nitrogen to the marine biosphere (100–200 Tg N year^−1^) (Codispoti, 2007; Grosskopf et al., 2012). Rates of N_2_ fixation can be estimated using a mass balance approach, for example, through deep-water nutrient stoichiometry (Gruber and Sarmiento, 1997; Deutsch et al., 2007) or natural signatures of stable isotopes (Montoya et al., 2002). Alternatively, N_2_ fixation can be measured indirectly with the acetylene reduction assay or directly with the ^15^N_2_ tracer assay (Zehr and Montoya, 2007 and references therein). Recently, it has been demonstrated that the commonly used ^15^N_2_ tracer assay leads to a significant underestimation of true N_2_ fixation rates, which may explain, at least partially, the apparent imbalance of sources and sinks of N in the global oceans (Grosskopf et al., 2012). In the former protocol of the ^15^N_2_ tracer assay, ^15−15^N_2_ gas was added directly as a bubble to water, and an instantaneous equilibrium between the ^15−15^N_2_ gas bubble and the N_2_ dissolved in water was assumed. Rates of N_2_ fixation were then calculated from the incorporation of ^15−15^N_2_ gas into biomass assuming a constant ^15^N-atom percent (atom%) in the dissolved N_2_ pool from the time of the tracer addition until the end of incubations (Montoya et al., 1996). The dissolution of a ^15−15^N_2_ gas bubble in water, however, is not instantaneous but time-delayed (Mohr et al., 2010). Consequently, in the past, rates of N_2_ fixation might have been underestimated depending on the incubation time, the timing of the injection of the ^15−15^N_2_ bubble relative to the diel cycle of organisms and the species composition of the diazotrophic community (Mohr et al., 2010; Grosskopf et al., 2012). To circumvent the potential underestimation of N_2_ fixation rates, a simple but effective twist has been proposed: the ^15^N tracer was added as an aliquot of ^15−15^N_2_-enriched seawater, which is prepared prior to incubations. Thereby, a constant ^15^N-atom% throughout incubations was ensured (Mohr et al., 2010).

These methodological advances stimulated a debate on how to prepare ^15−15^N_2_-enriched seawater—bearing in mind the practicability and time efficiency of the overall protocol. We took this debate as an inspiration, and tested methodological options to facilitate the preparation of ^15−15^N_2_-solutions and evaluated their benefits on the dissolution of ^15−15^N_2_ gas in water in relation to their time effort. We also estimated the risk of contaminations with trace elements (Fe, P, Mo, Mn, Zn, Cu) during the preparation of ^15−15^N_2_-enriched water, which may bias N_2_ fixation measurements in regions where N_2_ fixation is limited by the availability of P or Fe. Finally, recommendations are given for a rapid, simple and reliable procedure to enrich water with ^15−15^N_2_ gas.

## Materials and methods

### Efficiency of water degassing

**Figure 1:**
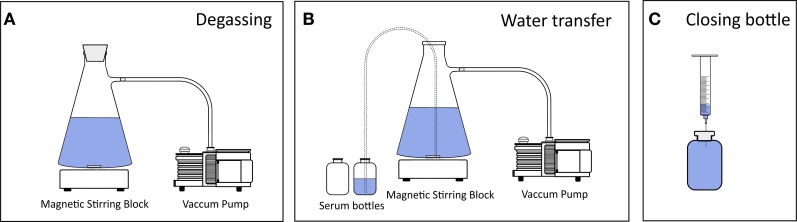
**Set-up of low-pressure degassing and bottling of water**. **(A)** Water was filled into a filtration flask, which was closed air-tight, placed on a magnetic stirring block and connected to a vacuum pump. During degassing the stirring was set to its maximum (1400 rpm) causing a turbulent vortex. **(B)** Water was transferred into borosilicate serum bottles via siphoning through gas-tight tubing so that atmospheric pressure forced the water to flow from the filtration flask into the serum bottles. Siphoning was initiated by air suctioning using a syringe. The tubing ends were kept at the bottoms of both flasks to avoid any water dripping and thus to minimize the gas–water interface. **(C)** The serum bottles were crimp-sealed headspace-free. A needle attached to a syringe without plunger was used as an outlet to not introduce air bubbles into the bottle while closing the bottle with a rubber stopper.

Two liters of deionized water were filled into a 4 L vacuum filtration flask suitable for low pressures. The flask was closed gas-tight, connected to a vacuum pump (Diaphragm Vacuum Pump N 026.3AN.18, KNF Neuberger GmbH, Freiburg, Germany) via gas-tight tubing (5 mm i.d.) and placed on a magnetic stirring block (Heidolph MR Hei-Mix L) (Figure 1). The maximum vacuum was 950 mbar below atmospheric pressure (atm. pressure) and water was mixed vigorously while degassing (magnetic stirring bar 40 × 8 mm, 1400 rpm). Degassing was conducted at 0, 200, 600, and 950 mbar below atm. pressure for up to 30 min. The stirring was stopped after the indicated degassing time and the pump switched off as soon as the water turbulence ceased. The water was transferred from the filtration flask into 160 mL borosilicate glass serum bottles using gas-tight tubing (transparent Tygon®, 8 mm o.d., 5 mm i.d.) via siphoning, i.e., atm. pressure was used to force water to flow from the filtration flask into a lower placed serum bottle. The tubing ends were positioned at the bottom of the serum bottle and filtration flask to limit the contact of water with air. As a measure of degassing efficiency, we determined the O_2_ concentration using a calibrated Clark-type microelectrode (tip diameter < 100 μm, Unisense A/S, Denmark) after the water was transferred into the serum bottles. The theoretical O_2_ concentration was calculated as

(1) $$\begin{array}{l} c_{O_{2}}=TGP\times \chi_{O_{2}}\times k_{\text{H}} \end{array}$$

where *TGP* is the total gas pressure (here equivalent to the absolute degassing pressure = standard atm. pressure—degassing pressure), χ_*O*2_ the mole fraction of O_2_ (= 0.2095) and *k*_H_ the Henry law constant (772.55 L atm mol^−1^ at 24°C, salinity 0).

### Preparation of ^15−15^N_2_-enriched seawater

The common procedure for all tests on the ^15−15^N_2_ dissolution was as follows: About 1.5 L of deionized water was degassed at 0 or 950 mbar low-pressure for 15 min as described above. The water was siphoned into 160 mL serum bottles and the bottles were crimp-sealed headspace-free with thick rubber stoppers (*h* = 1 cm) which can withstand over-pressure in the serum bottles (Figure 1). 2.5 mL of ^15−15^N_2_ gas (98 atom% ^15^N, Sigma-Aldrich) were injected into each bottle with a disposable needle (0.6 × 25 mm, Terumo Corporation, Leuven, Belgium) attached to a gas-tight syringe (2.5 mL, Luer Lock, SGE Analytical Science). Overpressure from the gas-tight syringe was released after withdrawing ^15−15^N_2_ gas from the gas cylinder. The solutions were vortex-mixed in the serum bottles for 1 min. Based on this procedure (unless stated differently in A–E or Table 1), we prepared 96 serum bottles which were grouped as triplicates and used to evaluate the effect of the following preparation steps on the ^15−15^N_2_ dissolution in water (see also Table 1):

**Table 1:** **Optional methodological steps which were tested for the preparation of ^15−15^N_2_-enriched water**.

**Methodological steps**		**Preparation of ^15−15^N_2_-enriched water**						
		**Degassing pressure [mbar]**	**Vol.^15−15^ N_2_ gas [mL]**	**Vol.water [mL]**	**Agitation**	**Water temp. during ^15−15^ N_2_ dissolution [°C]**	**Time of ^15−15^N_2_ dissolution [h]**	
Water degassing	0, 950 mbar	^*^	0–5	160	vortexed, 1 min	24	< 1	**Figure 3A**
Volume ^15−15^N_2_ gas	1, 1.25, 2.5, 4.5, 5.0, 7.0 mL	950	^*^	160	vortexed, 1 min	24	24	**Figure 3B**
Agitation	hand-shaking 30 s, vortex-mixing 1–20 min	950	5	160	^*^	24	< 1	**Figure 3C**
Compression of gas bubble	0.58 ± 0.10 → 0.07 ± 0.00 cm^3^	0, 950	5.0	160	vortexed, 1 min	24	24	**Figure 3D**
Water temperature	4°C, 24°C	950	5.0	160	hand-shaken, 30 sec	^*^	24	**Figure 3E**
Time of ^15−15^N_2_ gas dissolution	1 h, 24 h	950	2.5, 5.0	160	vortexed, 1 min	4 or 24	^*^	**Figure 3E**

* *tested parameter*.

- Water degassing: Water was degassed at 0 or 950 mbar low pressure prior to the addition of ^15−15^N_2_ gas. The volume of injected ^15−15^N_2_ gas varied between 0 and 5 mL per 160 mL water.
- Volume of ^15−15^N_2_ gas addition: The volume of the ^15−15^N_2_ gas ranged from 1 to 7 mL per 160 mL water.
- Agitation: The solutions were hand-shaken for 30 sec (50-fold vigorous inversion by 180°) or vortex-mixed for 1–20 min, respectively, after ^15−15^N_2_ had been added. During shaking, care was taken to disrupt the gas bubble into multiple small bubbles.
- Compression of injected gas bubble: A fraction of ^15−15^N_2_ will not dissolve in water but remain in the gaseous phase due to vapor–liquid equilibrium. Consequently, more ^15−15^N_2_ gas can be forced into the aqueous phase by reducing the bubble volume. We compressed the bubble volume by pressing 0.5 mL of water into half of the serum bottles after ^15−15^N_2_ gas had been injected. The subsequently added water was degassed at low-pressure when added to degassed water in the serum bottles, and not degassed when added to non-degassed water. The bubble volume was estimated by determining the bubble diameter with a ruler through the glass wall of the serum bottles.
- Storage time and temperature of the ^15−15^N_2_-enriched water: The solutions were stored for 1 or 24 h at 4°C (for 2.5 mL ^15−15^N_2_) or 24°C (for 2.5 and 5.0 mL ^15−15^N_2_ addition) after the ^15−15^N_2_ gas injection. In practice, the temperature of the ^15−15^N_2_ aliquot should equal the temperature of the incubation water. Thus, the water temperature was raised from 4°C back to 24°C in a water bath after the indicated storage time and before analyzing the ^15−15^N_2_ concentration.

Isotope ratios of dissolved N_2_ and concentrations of ^15−15^N_2_ were analyzed using either a membrane-inlet mass spectrometer (MIMS; GAM200, IPI) at the Max Planck Institute for Marine Microbiology in Bremen, Germany or a gas chromatography isotope ratio mass spectrometer (GC-IRMS; Thermo Delta V, Thermo Fisher Scientific Inc.) at the Stable Isotope Laboratories at the Department of Geology, Stockholm University, Sweden. For MIMS measurements, water was analyzed immediately and directly in each 160 mL serum bottle. For GC-IRMS measurements, subsamples from serum bottles were transferred to 12 mL Exetainer® using a wide needle (2 × 80 mm) and a 50 mL disposable syringe.

All tests on the ^15−15^N_2_ dissolution were conducted with deionized water. In order to test the validity of our results over a range of salinities, artificial seawater with a salinity of 0, 5, 20, and 35 was enriched with 5 mL ^15−15^N_2_ per 160 mL water. Generally, the solubility of gases decreases with elevated salinity. In our tests, this was confirmed by a negative linear correlation between the absolute ^15−15^N_2_ concentration and salinity (*R*^2^ = 0.9809). However, the final ^15^N-atom% excess was identical in saline and non-saline solutions because the relative solubility of ^14−14^N_2_, ^14−15^N_2_ and ^15−15^N_2_ decreased by the same magnitude at increasing salinity. Hence, our tests with deionized water are applicable for fresh- and seawater.

### Trace elements

Contaminations with trace elements can occur due to the contact of water with glass, plastic and rubber ware (Heinrichs and Hermann, 1990) or due to the use of artificial seawater as a solvent for ^15−15^N_2_ gas. We investigated whether trace elements accumulated during the preparation of ^15−15^N_2_-enriched water. All glass and plastic ware used in the above described experiments were washed with ultrapure HCl (10%, vol/vol), and rinsed and soaked in ultrapure water (Milli Q). HDPE-vials for trace element subsamples were preconditioned in 10% HCl overnight and thereafter rinsed with Milli Q water. During the preparation of ^15−15^N_2_-enriched water, 25 mL-subsamples were transferred into HDPE-vials (triplicates) after the following steps in the protocol: (step 1) vacuum degassing, (step 2) water transfer to serum bottles and 5 min of vortex-mixing, and (step 3) ^15−15^N_2_ gas injection and subsequent transfer of a ^15−15^N_2_ aliquot to the incubation volume. In addition, we prepared ^15−15^N_2_-enriched artificial seawater (S9883 Sigma) with a salinity of 35 to test whether artificial seawater can be an alternative to sterile filtered natural seawater, or whether it constitutes a substantial source of trace elements.

Trace element sub-samples were acidified with ultrapure HNO_3_ (Fisher Scientific) to a final concentration of 2% (vol/vol). Samples were analyzed for phosphorus (P), iron (Fe), molybdenum (Mo), manganese (Mn), zinc (Zn), and copper (Cu) using high-resolution inductively coupled plasma mass spectrometry (ICP-MS; Thermo Fisher Element II) at the ICBM, University of Oldenburg. ICP-MS measurements were done in medium resolution mode to separate molecular interferences from the analytes. The trace element concentrations were assessed with one-point calibration using single-element standards (Roth® or Sigma-Aldrich®) in the ppt (10^−12^) to lower ppb (10^−9^) range. Ultrapure 2% HNO_3_ (vol/vol) served as blank. The relative standard deviation was 2% for concentrations in the upper ppt to ppb range, and 15% (Fe, Cu, Mn, Zn) and 30% (Mo, P) for concentrations ≤ 10 ppt.

### Modified bubble injection of ^15−15^N_2_ assay

The addition of pre-prepared ^15−15^N_2_-enriched water may alter the trace element composition in the incubation water. To test an alternative and less invasive addition of ^15−15^N_2_ to an incubation volume, we combined the previously used bubble approach (Montoya et al., 1996) and the recently proposed dissolution approach (Mohr et al., 2010). Natural seawater samples were taken in the North Sea at different sampling stations and days. The seawater was filled headspace-free in 2 L polycarbonate bottles fitted with septum caps and the ^15^N tracer (98% + ^15−15^N_2_, Cambridge Isotope Laboratories, lot#I-17229) was added at a ratio of 2 mL ^15−15^N_2_ per liter of seawater. Bottles were gently mixed by hand for 15 min. Subsequently, the remaining gas bubble was removed in order to stop equilibration of N_2_ between the gas and aqueous phase. A water subsample was transferred into 12 mL Exetainer® and preserved with 100 μL of saturated HgCl_2_ solution for later analysis of the ^15−15^N_2_ concentration. After sub-sampling, the incubation bottles were refilled headspace-free with seawater to prevent any loss of ^15−15^N_2_ gas during the incubation.

### Application of the ^15−15^N_2_ dissolution assay

We incubated a culture of *Nodularia spumigena* KAC 12 (Karlberg and Wulff, 2013) by applying the dissolution (Mohr et al., 2010) and the bubble assay (Montoya et al., 1996). For the dissolution assay, water which was 0.2 μm-filtered (Isopore™ Membrane Filters, GTTP, Merck Millipore Ltd. Ireland) and degassed at 950 mbar low-pressure, was filled into 160 mL serum bottles, enriched with 2.5 mL ^15−15^N_2_ gas and vortex-mixed for 1 min. The seawater was taken in the Baltic Sea at station B1 (N 58° 48′ 18, E 17° 37′ 52). Triplicate incubations were initiated by adding 20 mL of the ^15−15^N_2_-enriched stock solution to 250 mL of *N. spumigena* suspension. Thereafter, the 250 mL serum bottles were crimp-sealed headspace-free. For the bubble assay, 300 μL of ^15−15^N_2_ were directly injected as a gas bubble to 250 mL *N. spumigena* suspension through the rubber stopper. The amount of ^15−15^N_2_ was calculated to give a similar ^15^N-atom% for both the bubble and the dissolution assay. All bottles were gently inverted 50 times by hand after the ^15−15^N_2_ addition, and incubated at 150 μE m^−2^ s^−1^ and 18°C for 0, 3, 6, 12, and 24 h. At the end of incubations, the following sub-samples were taken from each serum bottle: (1) Triplicate sub-samples were filled headspace-free into 12 mL Exetainer® vials to determine the ^15^N-atom% in the N_2_. These samples were preserved with 100 μL of saturated ZnCl_2_ solution. (2) 50 mL were preserved with Lugol's solution (L6146 Sigma) for cell counting of *N. spumigena*. The cell counting resulted in 7.4 ± 0.3 × 10^7^ cells L^−1^ (mean ± s.d., *n* = 9). (3) 150 mL were filtered onto pre-combusted GF/F filters and frozen at −80°C to quantify the amount of ^15−15^N_2_ incorporated into biomass. The GF/F filters were dried at 50°C overnight, pelletized into tin cups and analyzed on a Thermo Flash EA 1112 elemental analyzer coupled to an isotopic ratio mass spectrometer (Finnigan Delta Plus XP, Thermo Fisher Scientific) at the MPI, Bremen. Gases (calibrated against IAEA references) and caffeine were used as standards for the isotope ratios and the quantification of particulate organic carbon and nitrogen (POC, PON), respectively.

N_2_ fixation rates were calculated as

(2) $$\begin{array}{l} N_{2}\;fixation\;\;rate=\frac{\left(A_{sample}^{PN}-A_{control}^{PN}\right)}{\left(A_{N2}-A_{control}^{PN}\right)}x\frac{\left[PN\right]}{\Delta t} \end{array}$$

where *A* is the ^15^N-atom% in the dissolved N_2_ pool (*A*_*N*_2__) and particulate material (*A*^*PN*^), and [*PN*] is the concentration of particulate material. A significant ^15^N-uptake other than ^15^N_2_ fixation due to the impurity of ^15^N_2_ gas (Dabundo et al., 2014) could be excluded for our studies since traces of ^15^${\text{NH}}_{4}^{+}$, ^15^${\text{NO}}_{3}^{-}$, or ^15^${\text{NO}}_{2}^{-}$ in the ^15−15^N_2_-enriched water were not detectable using GC-IRMS.

The parameter *A*_*N*_2__, as given in Equation (2), is defined as ^15^N-atom% in the dissolved N_2_ pool, that is, the sum of naturally abundant and added ^15^N. However, in the literature, *A*_*N*_2__ is occasionally denoted as ^15^*N-enrichment* or ^15^*N-excess enrichment*. This misleading use of terms may lead to miscomprehension and we recommend a clear distinction between ^15^N-atom% and ^15^N-atom% excess in future studies.

## Results and discussion

### Efficiency of degassing

Recent studies applied water degassing at 750–960 mbar below atm. pressure for approximately 1 h, and degassing set-ups were similar to the one shown here (Figure 1) or a membrane flow-through system was used (Mohr et al., 2010; Grosskopf et al., 2012; Rahav et al., 2013). Alternatively, ambient air was removed by purging with helium (Wilson et al., 2012). Nevertheless, the benefit of the degassing pressure/duration on the degassing efficiency and finally on the actual ^15−15^N_2_ dissolution has not been assessed systematically.

**Figure 2:**
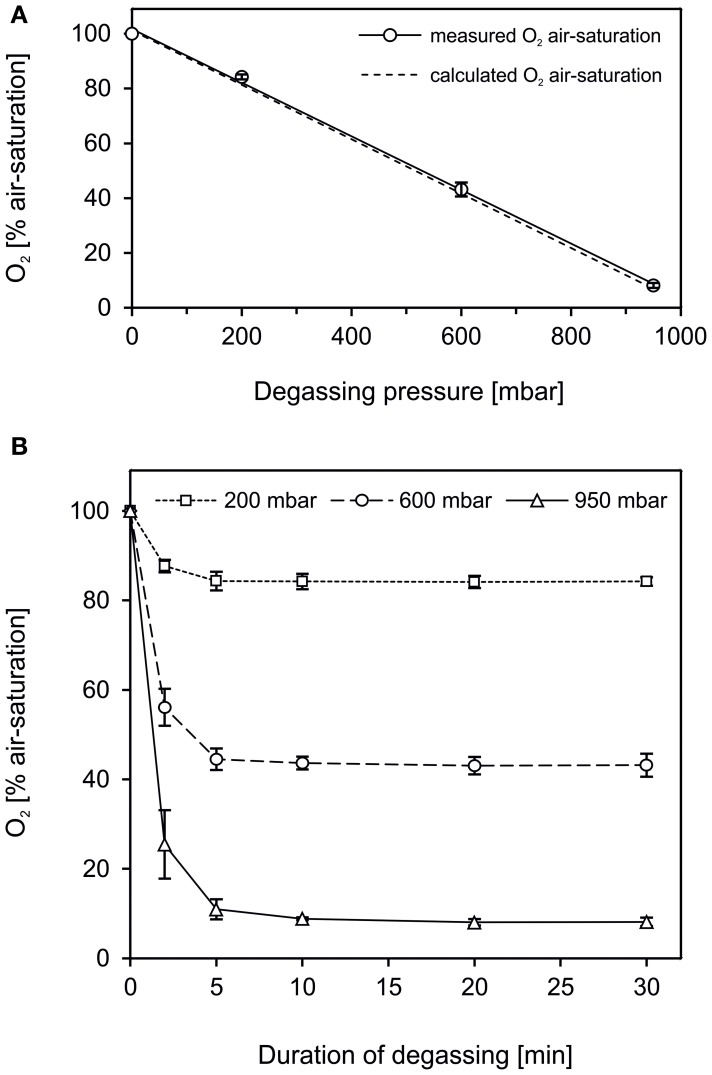
**The efficiency of water degassing conducted in a set-up as shown in Figure 1**. **(A)** Deionized water was degassed at 0, 200, 600, and 950 mbar below atmospheric pressure. The level of gas removal, determined as O_2_ air-saturation, was positively correlated to the degassing pressure (straight line, R^2^ = 0.9985). The efficiency of the degassing procedure was high and met the theoretical O_2_ air-saturation at a given low-pressure calculated according to Henry's law (dashed line). **(B)** The gas removal progressed fast, with the major part of gas being removed within the first 2–5 min. **(A,B)** Data are given as mean ± s.d. (*n* = 6).

The solubility of gas follows Henry's law, i.e., the amount of gas dissolved in a liquid is proportional to its partial pressure.

(3) $$\begin{array}{r} p=c\times k_{\text{H}} \end{array}$$

where *p* is the partial pressure, *c* the concentration of the solute and *k*_*H*_ the Henry law constant. In agreement, in our tests the degree of degassing and the degassing pressure were positively correlated (Figure 2A, *R*^2^ = 0.9985). The efficiency of the degassing set-up to remove dissolved gas from water was high and met theoretical assumptions. Following Equation (1), water degassing at 200, 600, and 950 mbar low-pressure reduces the O_2_ air-saturation to 83.0, 42.2, and 6.5%, respectively. By applying the set-up as shown in Figure 1, dissolved gas was removed from water to 84.2 ± 0.9% O_2_ air-saturation (mean ± s.d., *n* = 6) at 200 mbar, 43.0 ± 1.9% (*n* = 6) at 600 mbar and 8.0 ± 0.7% (*n* = 6) at 950 mbar low-pressure. The minor difference between the measured and theoretical O_2_ air-saturation can be explained either by a deviation of the actual atm. pressure from the standard atm. pressure or by O_2_ contamination as the water was transferred from the filtration flask to the serum bottles. Degassing was accelerated by vigorous stirring and the maximum gas removal achieved after 5–10 min (Figure 2B). Hence, degassing at 950 mbar low-pressure in a simple set-up as applied here is an efficient and fast way of gas removal yielding a high degree of gas removal to ≤10% air-saturation within minutes (Figure 2B).

### Preparation and evaluation of ^15−15^N_2_-enriched water

#### The effect of water degassing on the dissolution of ^15−15^N_2_ gas

**Figure 3:**
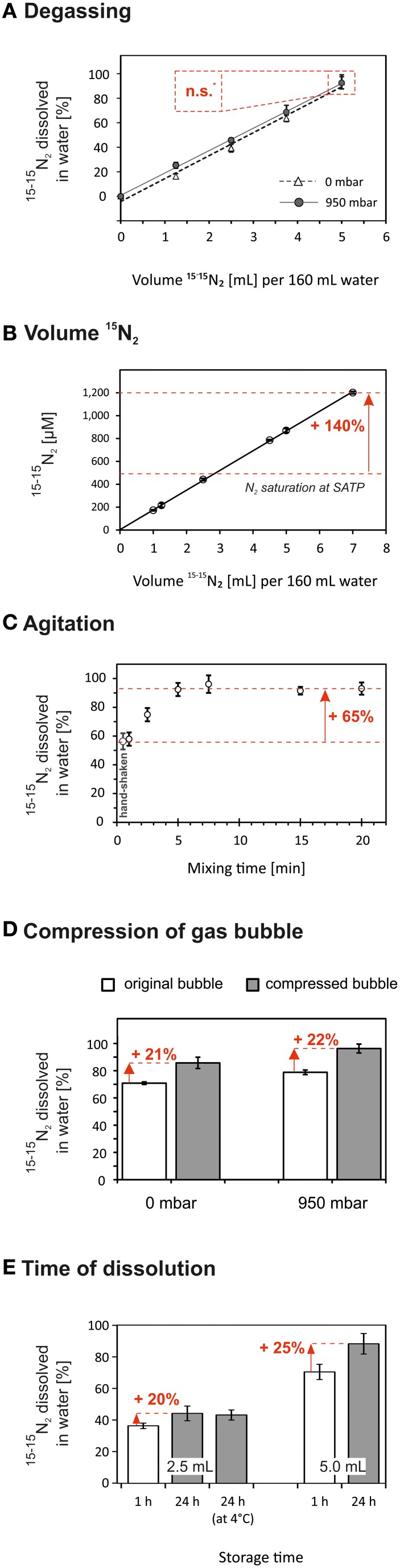
**The dissolution of ^15−15^N_2_ in water could be accelerated by applying optional methodological steps during the preparation of ^15−15^N_2_-enriched water**. Red numbers indicate the relative increase of the amount of ^15−15^N_2_ gas which dissolved in the water after the optional steps have been applied. For each treatment the highest amount of ^15−15^N_2_ dissolved in water was normalized to 100% (except **B**). Data are given as mean ± s.d. (*n* = 3). **(A)** The amount of ^15−15^N_2_ dissolved in water was not significantly (n.s., *p* > 0.05) different in water degassed at 0 or 950 mbar below atmospheric pressure. Nonetheless, the ^15^N-atom% in the final incubation volume can be increased by two indirect effects of water degassing on the final ^15^N-atom% excess (data not shown, see Section The effect of water degassing on the dissolution of ^15−15^N_2_ gas). **(B)** The ^15−15^N_2_ concentration was positively correlated to the volume of injected ^15−15^N_2_ gas even at N_2_ oversaturation. The N_2_ solubility at standard ambient temperature and pressure (SATP) is indicated by the lower dashed line (Colt, 2012). **(C)** Agitation was an effective mode to raise the ^15−15^N_2_ dissolution in water. The maximum ^15^N-tracer concentration was achieved after 5 min of vortex mixing. **(D)** The injected ^15−15^N_2_ gas bubble was compressed by the addition of water into the bottle, i.e., pressure increase in the bottle. The volume reduction of the gas bubble enhanced the amount of ^15−15^N_2_ dissolved in water by 21–22%. **(E)** Solutions of ^15−15^N_2_ were stored at 24°C or 4°C for 1 h or 24 h. After 24 h the ^15−15^N_2_ dissolved in water increased by 20–25% as compared to 1 h storage time. A colder storage temperature had no significant effect on the ^15−15^N_2_ concentration.

Gases and their isotopes have a similar solubility (Klots and Benson, 1963), and the N_2_ solubility is linearly correlated to the partial pressure (Equation 3). Correspondingly, our data showed that water degassing had no *direct* effect on the amount of ^15−15^N_2_ which dissolved in water after ^15−15^N_2_ had been added (Figure 3A). Nonetheless, water degassing had two *indirect* effects increasing the ^15^N-atom% excess in the final incubation volume. Firstly, degassing at 950 mbar low-pressure lowered the ^14−14^N_2_ and ^14−15^N_2_ background in the ^15−15^N_2_-enriched water to ≤10% (compare with Figure 2). Thereby, less ^14^N is added together with ^15−15^N_2_-enriched water to the incubation volume and the final ^15^N-atom% excess in the incubation volume would increase by 4.5% (e.g., from 5.0 to 5.2% assuming that the aliquot of ^15−15^N_2_-enriched water equals 5% of the final incubation volume). Secondly, water degassing lowered the initial total gas pressure and thus more ^15−15^N_2_ could be added to under-saturated water without risking the borosilicate serum bottles to explode. Roughly, the serum bottles could withstand the overpressure of a maximum of 5 mL ^15−15^N_2_ per 160 mL non-degassed water and 7 mL ^15−15^N_2_ per 160 mL of 950 mbar-degassed water. This can yield an additional increase in the final ^15^N-atom excess of 30% (e.g., from 5.2 to 6.8% ^15^N-atom% excess).

#### The effect of the volume of injected ^15−15^N_2_ gas on the ^15−15^N_2_ concentration

In agreement with Equation 3, we found a strong positive correlation between the amount of injected ^15−15^N_2_ gas and the measured ^15−15^N_2_ concentration in water even when exceeding the standard solubility of N_2_ in water (*R*^2^ = 0.9998, Figure 3B). The maximum ^15−15^N_2_ concentration was 1200 μmol L^−1^, that is, adding a 20 mL aliquot of this solution to 1 L of incubation volume (24°C, 0 PSU) would be sufficient to achieve a final ^15^N-atom excess of 5%. Further, instead of serum bottles, we tested the usage of Exetainer® vials as water containers. We injected 2 mL of ^15−15^N_2_ gas into a 12 mL Exetainer® filled with non-degassed water and vortex-mixed the solution for 1 min. The ^15−15^N_2_ concentration was 782 ± 48 μmol L^−1^ (*n* = 3), i.e., an aliquot of 30 mL added to 1 L of incubation volume (24°C, 0 PSU) would be sufficient to achieve a final ^15^N-atom excess of 5%. The preparation of ^15−15^N_2_-enriched water in the Exetainer® vials was fast but the overpressure in the vials was high which made it difficult to press the total volume of ^15−15^N_2_ gas into the vials. Moreover, the ^15−15^N_2_ recovery was low (around 11%), presumably because a large gas bubble remained even after vortex-mixing.

#### The effect of agitation on the dissolution of ^15−15^N_2_ gas

In general, agitation promotes turbulent diffusion of gases across the gas–water interface. Hand shaking and vortex mixing had the same efficiency on the ^15−15^N_2_ dissolution relative to the mixing time; however, due to practical reasons we applied vortex-mixing in cases when the mixing exceeded 30 s. Vortex mixing yielded the maximum ^15−15^N_2_ concentration within 5 min. The amount of ^15−15^N_2_ which dissolved in water could therefore be increased by 65% after 5 min of vortex mixing compared to the ^15−15^N_2_ dissolution after 30 s of hand-shaking (Figure 3C).

#### The effect of compressing the gas bubble on the dissolution of ^15−15^N_2_ gas

Dinitrogen gas is rather insoluble in water whereby a major part of N_2_ remains in the gas phase. Thus, a reduction of the bubble volume by increasing the pressure inside the serum bottle should enhance the amount of ^15−15^N_2_ which dissolves in water. We injected 5 mL ^15−15^N_2_ gas to 160 mL non-degassed (0 mbar) and degassed water (950 mbar) which created a bubble of 0.58 ± 0.10 cm^3^ (mean ± s.d., *n* = 12). After pressing 0.5 mL of water into half of the serum bottles, the bubble size was compressed to 0.07 ± 0.00 cm^3^ (*n* = 6) irrespective of the earlier applied degassing pressure. This bubble compression led to a rise in ^15−15^N_2_ by 21–22% (Figure 3D). In fact, this rise in ^15−15^N_2_ corresponded to the amount of ^15−15^N_2_ which was initially trapped in the bubble but dissolved in the liquid as the bubble was compressed. Compressing the gas bubble was a quick process but occasionally the pressure increase within the serum bottle caused the bottle to shatter risking the applicants' health.

#### The effect of storage time and temperature on the dissolution of ^15−15^N_2_ gas

The amount of ^15−15^N_2_ which dissolved in water increased by 20–25% after 24 h compared to 1 h storage (Figure 3E). Although this was a substantial effect, this approach to increase the ^15−15^N_2_ concentration seemed inefficient considering its time effort. Decreasing the temperature during storage from 24°C to 4°C did not yield higher ^15−15^N_2_ concentrations. Although gases are more soluble at colder temperatures, the re-warming of the ^15−15^N_2_ solution from 4°C to 24°C before the ^15−15^N_2_ concentration analysis might have reversed the effect of the colder storage temperature; however, this step is inevitable to not alter the temperature in the incubation volume.

#### Final ^15^N-atom% excess using the dissolution approach

For most field studies an incubation volume of 1–4 L and a final ^15^N-atom excess of 2–5% is applied (Grosskopf et al., 2012 Supplementary information). We achieved a ^15^N-atom excess of 5% in the final incubation volume by applying the following steps: 5 mL of ^15−15^N_2_ gas were added to 160 mL water (degassed at 950 mbar low-pressure for 15 min) in serum bottles. Thereafter, the ^15−15^N_2_ solution was vortex-mixed for 1 min. We transferred 50 mL of the ^15−15^N_2_ stock solution to a 1 L Schott glass bottle which was filled to the brim (1150 mL), i.e., the volume of the ^15−15^N_2_ aliquot equaled less than 5% of the total incubation volume. The final ^15^N-atom% excess was 4.8 ± 0.1% (*n* = 10) with no significant difference after 0.5 and 12 h following the ^15−15^N_2_ aliquot addition (*p* > 0.05). The ^15^N-atom% excess might have been further enhanced by increasing the initial ^15−15^N_2_ gas addition to 7 mL ^15−15^N_2_ per 160 mL water and prolonging vortex-mixing to 5 min. During our later tests, the addition of 7 mL ^15−15^N_2_ gas to 160 mL degassed water yielded a ^15−15^N_2_ concentration of 1200 μmol L^−1^ (Figure 3B). Based on these results, only 2% of the incubation volume (24°C, salinity 0) would have to be replaced with ^15−15^N_2_-enriched water to reach a final ^15^N-atom excess of 5%. Importantly, the amount of ^15−15^N_2_ that is transferred to the incubation water has to be adjusted according to the standard N_2_ solubility at a given water salinity and temperature.

We validated whether the transfer of ^15−15^N_2_-enriched water from the incubation bottle to an Exetainer® vial may cause a loss of ^15−15^N_2_ to the atmosphere. The ^15−15^N_2_-enriched water from an incubation bottle was gently aspirated with a 50 mL syringe (Plastic Sterile Plastipak) and expelled through a 0.2-μm syringe filter to the bottom of an Exetainer® vial. The gas–water interface was minimized during the water transfer by not dripping the solution into the Exetainer® vial, but using a needle (2 × 80 mm) or small tubing as extension of the syringe to gently release the water to the bottom of the vial. The ^15−15^N_2_ concentration was measured directly in the incubation volume and after a sub-sample was transferred to an Exetainer® using MIMS. On average the relative ^15−15^N_2_ loss was 4 ± 1% (mean ± s.d., *n* = 6) after the above described sample transfer.

#### Final ^15^N-atom% excess using the modified ^15−15^N_2_ bubble addition

The addition of 2 mL ^15−15^N_2_ gas per liter seawater and subsequent removal of the bubble in the incubation volume yielded a ^15^N-atom excess of 6.6 ± 1.7% (mean ± s.d., *n* = 12) ranging from 3.9 to 10.1% for water with salinities from 25 to 0.4. This wide range was driven by three bottles with considerably lower (3.9%) or higher (10.1%) values, but in nine of the 12 bottles the ^15^N-atom excess was 6.8 ± 0.9%. When using the modified bubble addition, a consistent agitation should be used to ensure a small variability of ^15^N-atom% among experiments. One disadvantage of this approach is the less efficient use of ^15−15^N_2_ gas (utilizing 40% of the ^15−15^N_2_ gas compared to 70% when adding ^15−15^N_2_-enriched water as a stock solution). Nonetheless, the modified bubble approach is a less invasive approach for N_2_ fixation measurements compared to the addition of pre-prepared ^15−15^N_2_-enriched stock solutions and may minimize the risk of trace element contaminations (see Section Trace Elements).

### Trace elements

Trace element concentrations in the ^15−15^N_2_-enriched water were ≤2 nmol L^−1^ for Fe, P, Mn, Mo, Cu, Zn. The individual element concentrations in the final incubation volume increased by a maximum of 0.01 nmol P L^−1^, 0.1 nmol Fe L^−1^, 0.04 nmol Mn L^−1^, 0.1 nmol Cu L^−1^, and 0.09 nmol Cu L^−1^. These concentrations were calculated by assuming that the volume of the ^15−15^N_2_-enriched water which is transferred to the incubations equals 5% of the total incubation volume. Concentrations of Mo did not differ in the ^15−15^N_2_-enriched water and blank solutions. These trace element contaminations should be seen as an estimate. We expect that contaminations vary depending on the specific material used during the preparation of ^15−15^N_2_-enriched water and also the history of that material. In our tests, major contamination sources for Fe were the contact of water with glass ware and colored rubber stoppers. In contrast, the utilization of stainless steel needles (0.6 × 25 mm, Terumo Corporation, Leuven, Belgium), which were used for the ^15−15^N_2_ gas injection into the serum bottles, led to no substantial Fe-contamination because the cannulas were by default covered with silicone (personal communication with the supplier TERUMO BCT Europe).

The artificial seawater (salinity of 35) was highly enriched with trace metals, ranging from 23–70 nmol L^−1^ for Fe, Mn, Cu, Zn. Levels of Mo were low, 0.1 nmol L^−1^, and levels of P were not different from blank levels. These concentrations confirmed the declaration of the manufacturer Sigma stating their sea salt S9883 may contain ≤500 nmol L^−1^ of trace elements (information received after written request). The usage of this specific artificial seawater would have led to trace metal concentrations in the incubation water of 3.5 nmol L^−1^ for Fe, 2.3 nmol L^−1^ for Mn, 2.9 nmol L^−1^ for Cu, 1.2 nmol for Zn and 5 pmol L^−1^ for Mo assuming that the volume of the artificial seawater added to the incubation volume equals 5 Vol%. Such concentration levels are in excess of those in wide regions of the marine environment. Artificial seawater prepared with sea salt of a different batch, supplied by manufacturers other than Sigma (S9883) or seawater prepared using ultrapure single element powder may yield lower (or higher) concentrations of trace elements.

### Application of ^15−15^N_2_ dissolution assay

**Figure 4:**
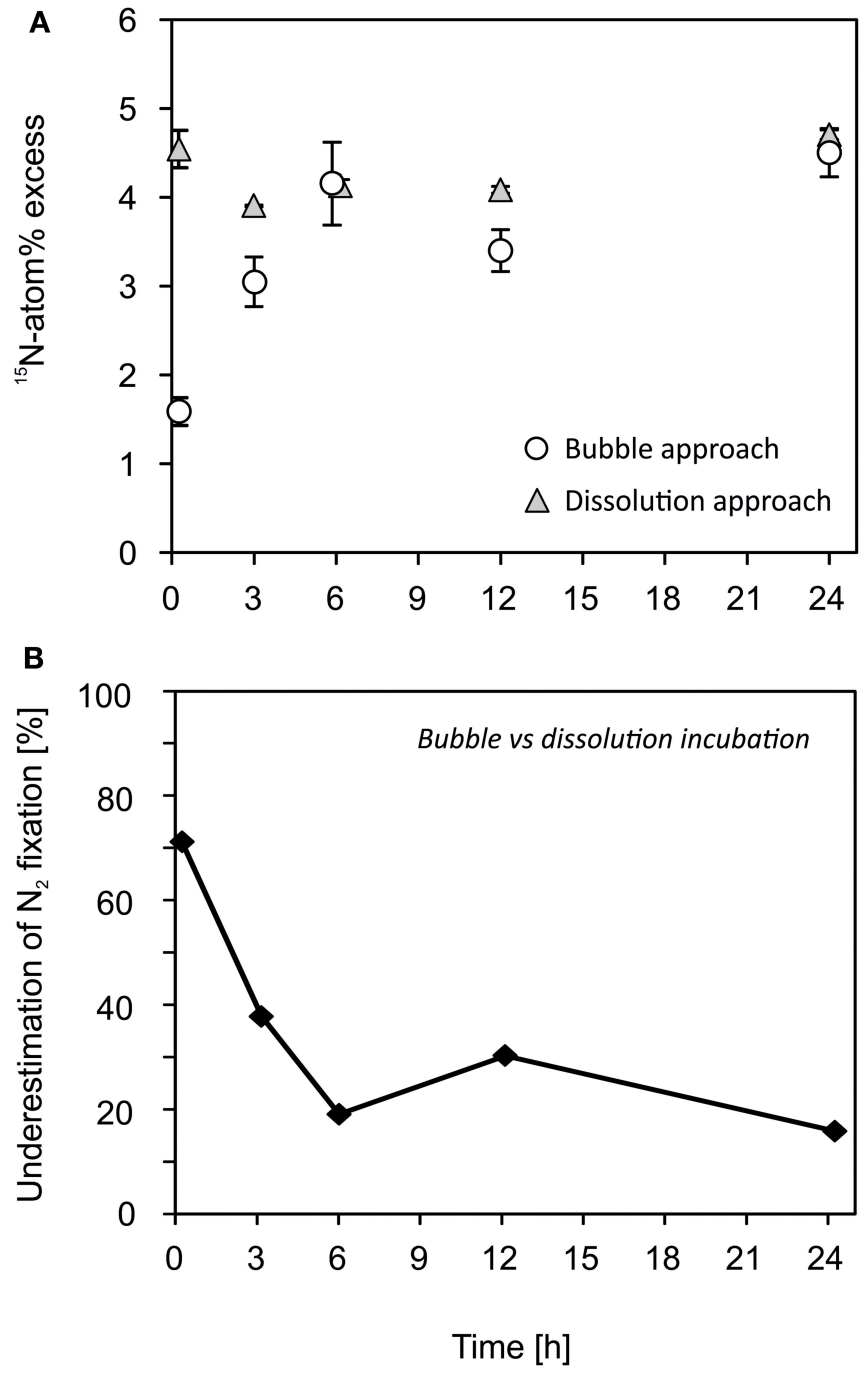
**A ***Nodularia spumigena*** culture was incubated following the former bubble-addition and the modified dissolution assay**. **(A)** The dissolution of a ^15−15^N_2_ gas bubble was time-dependent. The ^15^N-atom% increased over time if the ^15^N-tracer was added as a bubble directly to the incubation volume (see also Mohr et al., 2010). The ^15^N-atom% was more consistent as an aliquot of ^15−15^N_2_-enriched water was added to the *N. spumigena* solution (dissolution approach). **(B)** Rates of N_2_ fixation were underestimated by 16–71% when using the bubble approach compared to the dissolution approach.

The dissolution approach attained a ^15^N-atom% excess of 4.3 ± 0.3% (mean ± s.d., *n* = 35). The differences between the time points were low and are explained by differences of the initial ^15−15^N_2_ stock solution as we used a different serum bottle of the ^15−15^N_2_ stock solution for each set of triplicates of each time point. In contrast, the ^15^N-atom% excess increased from 1.6 ± 0.1% (*n* = 9) after 15 min to 4.5 ± 0.3% (*n* = 9) after 24 h when the ^15−15^N_2_ gas was injected as a bubble (Figure 4A). This led to an underestimation of N_2_ fixation by 38% after 3 h and 16% after 24 h incubations if the ^15^N-atom% was assumed to be 4.5% throughout the entire incubation period (Figure 4B). The decrease in underestimation of N_2_ fixation with time was due to the fact that the ^15−15^N_2_ reached its vapor–liquid equilibrium after approximately 6 h (Figure 4B). In addition, the positively buoyant colonies of *Nodularia spumigena* floated and assembled close to the gas bubble where they were exposed to a higher ^15^N-atom% compared to organisms more distant from the gas bubble. This might have compensated for the initially lower ^15−15^N_2_ concentration in water (see also Grosskopf et al., 2012 SI; White, 2012).

## Recommendations and comments

The following guidelines are recommended as best practice for an efficient and reproducible preparation of ^15−15^N_2_-enriched water and the reliable determination of N_2_ fixation rates:

1. For the preparation of ^15−15^N_2_-enriched water, we suggest to add ^15−15^N_2_ to sterile-filtered water in excess of the standard N_2_ solubility and to mix the solution vigorously, preferably by vortex-mixing for at least 5 min. Prior to the addition of ^15−15^N_2_, sterile-filtered water can be degassed at low-pressure (≥950 mbar) to indirectly increase the ^15−15^N_2_-atom% excess in the final incubation volume (see Section The effect of water degassing on the dissolution of ^15−15^N_2_ gas). The protocol for the ^15−15^N_2_ enrichment, however, might be adjusted according to the experimental set-up, time plan and study area. A rating according to the benefits and drawbacks of the methodological steps for the preparation of ^15−15^N_2_-enriched water is given in Table 2.
2. Natural seawater from the specific sampling stations/depths should be used rather than artificial seawater in order to avoid trace element contaminations. In addition, to minimize possible contaminations during the preparation of ^15−15^N_2_-enriched water, we recommend washing all equipment with 10% HCl (vol/vol) followed by several rinses with MilliQ, and to use old and worn glass ware which has lost its element impurities. Transparent rubber stoppers and tubing (for example, transparent PVC, PE or Tygon®, which are N-free) are preferable over colored rubber stoppers and tubing. In our tests, the mean concentration of trace elements in the incubation volume was estimated to increase by up to 0.1 nmol L^−1^ due to the addition of pre-prepared ^15−15^N_2_-enriched water. We consider trace element contamination levels of up to 0.1 nmol L^−1^ as minor for many regions of the aquatic environment. Yet, marine pelagic (cyanobacterial) N_2_ fixation can be limited by the availability of Fe and P, especially in the open ocean (e.g., Sanudo-Wilhelmy et al., 2001; Mills et al., 2004; Turk-Kubo et al., 2012). We therefore advise against the preparation of ^15−15^N_2_ enriched water as shown in Figure 1 when working in regions where concentrations of dissolved Fe are very low (≤0.1 nmol L^−1^), e.g., the South Atlantic Ocean or parts of the central North and South Pacific Gyres (Brown et al., 2005; Sohm et al., 2011). Here, the modified bubble addition approach is recommended due to a lower risk of trace element contamination.
3. A sub-sample of the incubation water should be taken for MIMS or GC-IRMS analyses in order to determine the actual enrichment of ^15−15^N_2_ and to ensure the accurate calculation of N_2_ fixation rates. When using the modified bubble method, every individual incubation bottle should be sub-sampled. We further recommend that ^15^N_2_ gas bottles are checked for potential impurities with ^15^N-compounds other than ^15^N_2_ (Dabundo et al., 2014).

**Table 2:** **Rating of optional methodological steps for the preparation of ^15−15^N_2_-enriched water according to their positive (••) or negative (◦◦) effects on parameters which are of importance for N_2_ fixation assays**.

**Methodological steps**	**Time effort**	**Final ^15^ N-atom% excess**	**Volume of ^15−15^N_2_ aliquot added to incubation volume**	**Accuracy ^15^ N-atom% excess**	**Utilization of ^15−15^ N_2_ gas**	**Trace element concentration**
Water degassing	◦	•	•	•◦	•◦	◦
Volume ^15−15^N_2_ gas	••	••	••	•◦	•◦	•◦
Agitation	••	••	••	•◦	••	◦
Compression of gas bubble	•	•	•	◦	•	•◦
Water temperature	◦◦	◦	•	•◦	•	n/a
Time of ^15−15^N_2_ gas dissolution	◦◦	•	•	•◦	•	n/a
Modified ^15−15^N_2_ bubble addition	•	•	–	•	◦	••

*•• very good • good •◦ no effect ◦ acceptable ◦◦ not acceptable (n/a = not available)*.

**Figure 5:**
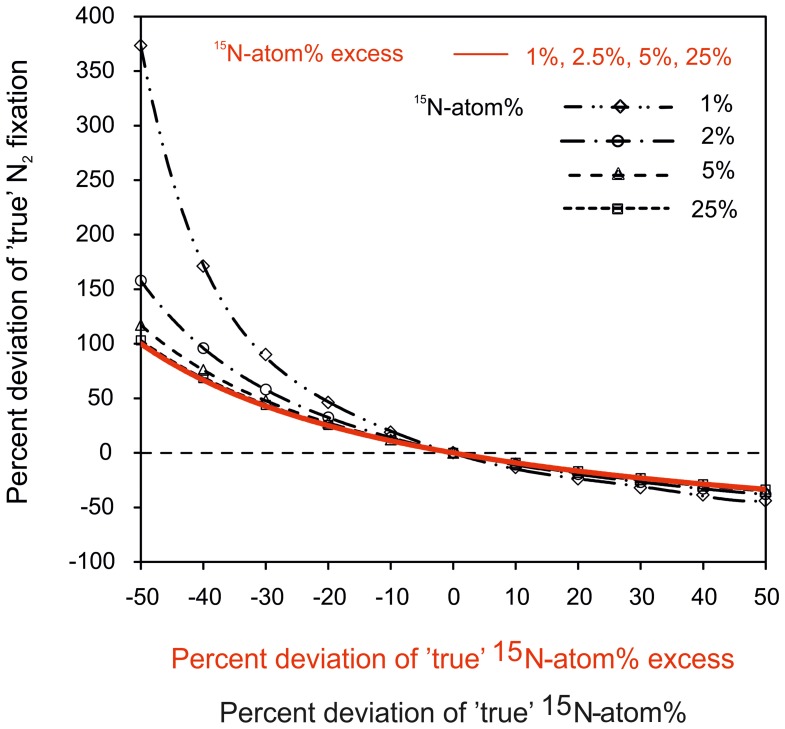
**Modeled deviation from ***true***, actual N_2_ fixation rates in correlation to the potential deviation from ***true*** values of ^15^N-atom% (in black) or ^15^N-atom% excess (in red)**. Following Equation 2, a deviation of ^15^N-atom% or ^15^N-atom% excess from the *true* value will lead to significant under- or overestimations of N_2_ fixation. This error in N_2_ fixation rates is especially pronounced when the ^15^N-atom% or ^15^N-atom% excess is underestimated. In addition, the variable ^15^N-atom% is corrected for the naturally present ^15^N (0.366%) for calculations of N_2_ fixation rates. This correction has a more pronounced weighting on the calculated N_2_ fixation when using a low ^15^N-atom% of ≤2% compared to a higher ^15^N-atom of ≥5%. The percent deviation was calculated as difference between the *incorrect*, i.e., falsely estimated value of ^15^N-atom%/^15^N-atom% excess and their *true* values.

To highlight the importance of the determination of the ^15^N-atom% in the incubation, we modeled the effect of under-/overestimating the ^15^N-labeling on rates of N_2_ fixation (Figure 5). Rates of N_2_ fixation are calculated according to Equation (2) and an incorrect assumption of the ^15^N-atom% or ^15^N-atom% excess leads to a significant bias in N_2_ fixation rates. The percent deviation from true N_2_ fixation rates is more pronounced if the ^15^N-atom% or ^15^N-atom% excess is underestimated compared to occasions when it is overestimated. Moreover, the magnitude of potential errors in N_2_ fixation rates is lower when using a ^15^N-atom% of ≥5% compared to a lower ^15^N-atom% of ≤2% (Figure 5). A high ^15^N-atom% should also be used in regions with high biomass and/or high productivity, but low N_2_-fixing activity. Here, a high ^15^N-atom% increases the detection limit of N_2_ fixation since the ^15^N-PON signal from N_2_ fixation can be attenuated by the presence of non-diazotroph PON.

Currently, the ^15^N_2_ tracer assay is the only available method for a direct assessment of N_2_ fixation by aquatic diazotrophs and its application has greatly advanced our understanding of the global N-cycle. Nevertheless, the systematic underestimation of actual N_2_ fixation rates by the former bubble approach demanded the development and evaluation of a revised protocol. In the future, the application of a reliable ^15^N_2_ tracer assay will be of importance to advance our knowledge in diazotrophic activity and biogeography in the aquatic environment. Especially, the activity of unicellular and symbiotic diazotrophs is believed to have been underestimated up to 6-fold during past field campaigns (Grosskopf et al., 2012). A modified, standardized N_2_ fixation methodology is therefore expected to yield higher rates of N_2_ fixation which may, at least partially, resolve the discrepancy between N-gain and -loss processes in marine N budget calculations (Codispoti, 2007).

## Author contributions

IK, GL, JD, WM and HP designed the study. IK, GL, PB, HM, JD and WM conducted the experiments and sample analyses. All authors contributed to the interpretation of data and drafting the work.

### Conflict of interest statement

The authors declare that the research was conducted in the absence of any commercial or financial relationships that could be construed as a potential conflict of interest.
